# Realistic Ultrasound Simulations of Healthy and Osteoarthritic Cartilage

**DOI:** 10.3390/jimaging12080379

**Published:** 2026-08-12

**Authors:** Roby Weeteling, Yuexin Qi, Rob P. A. Janssen, Keita Ito, Corrinus C. van Donkelaar, Richard G. P. Lopata, Min Wu

**Affiliations:** 1 Photoacoustics and Ultrasound Laboratory Eindhoven (PULS/e), Department of Biomedical Engineering, Eindhoven University of Technology, 5612 AZ Eindhoven, The Netherlands; y.qi1@tue.nl (Y.Q.); r.lopata@tue.nl (R.G.P.L.); m.wu@tue.nl (M.W.); 2 Orthopaedic Biomechanics, Department of Biomedical Engineering, Eindhoven University of Technology, 5612 AZ Eindhoven, The Netherlands; r.p.a.janssen@tue.nl (R.P.A.J.); k.ito@tue.nl (K.I.); c.c.v.donkelaar@tue.nl (C.C.v.D.); 3 Department of Orthopaedic Surgery & Trauma, Máxima Medical Center Eindhoven/Veldhoven, 5631 BM Eindhoven, The Netherlands; 4 Value-Based Health Care, Faculty of Paramedical Sciences, Fontys University of Applied Sciences, 5612 MA Eindhoven, The Netherlands

**Keywords:** articular cartilage, osteoarthritis, ultrasound imaging, computational modeling, microstructural model

## Abstract

Osteoarthritis (OA) causes irreversible cartilage damage, highlighting the need for early and sensitive assessment. Current imaging modalities are limited in detecting early-stage changes. Ultrasound (US) provides a non-invasive and accessible alternative, but its clinical adoption is limited by the lack of standardized protocols and reliable cartilage assessment. Simulations can be used to address these challenges by enabling system design, acquisition optimization and validation by providing ground truth when in vivo ground truth is unavailable. The aim of this study is to develop an in silico framework for realistic US imaging of healthy and OA cartilage by combining accurate acoustic wave modeling with a 2D microstructural cartilage phantom. The model was calibrated to healthy cartilage using first-order speckle statistics and extended to simulate degeneration through changes in structural and acoustic properties. As a proof-of-concept study, simulations were evaluated against limited ex vivo US data from healthy and OA cartilage and compared with literature data. The simulations reproduced key OA-related features and trends, including changes in reflection coefficient (R), integrated reflection coefficient (IRC), apparent integrated backscatter (AIB), and gray level distributions. These findings demonstrate the feasibility of using microstructure-based tissue phantoms to model healthy and OA cartilage. The framework provides a platform for systematic investigation of cartilage microstructure and US-derived features and may support future generation of synthetic datasets for data-driven and AI-based OA assessment.

## 1. Introduction

Osteoarthritis (OA) is a prevalent joint disease characterized by disrupted tissue homeostasis, with compromised cartilage structure and integrity as key features [1]. Articular cartilage is a specialized connective tissue that provides a durable, low-friction surface and distributes load to the subchondral bone, thereby preserving joint function. It consists mostly of an extracellular matrix (ECM), primarily consisting of collagen and proteoglycans, within which sparse chondrocytes are organized in small units known as chondrons, i.e., cells surrounded by a thin layer of a softer pericellular matrix (PCM). These structural components are organized into distinct depth-dependent zones with characteristic collagen fiber orientation, proteoglycan content, and chondron morphology, which collectively determine the unique properties of cartilage [1]. Unfortunately, the avascular nature limits its regenerative capacity. Consequently, current treatments mainly focus on pain relief, with joint arthroplasty reserved for advanced stages. Timely interventions, such as lifestyle modifications and joint-preserving therapies, can slow disease progression. Therefore, accurate and sensitive assessment of cartilage damage is essential for effective OA management [2,3].

Unfortunately, current imaging methods struggle to detect cartilage damage at an early stage, when microscopic changes occur before noticeable symptoms: (1) X-ray does not visualize cartilage, (2) clinical MRI is costly with insufficient microscopic resolution, and (3) arthroscopy is invasive and depends on subjective visual grading [4]. In contrast, ultrasound (US) offers a non-invasive, fast and affordable imaging modality that is widely available for point-of-care use. Numerous US techniques have been developed for accurate and sensitive assessment of cartilage damage, improving OA classification, and supporting early detection, real-time monitoring, and treatment evaluation [2,5]. Quantitative US metrics can indicate surface fibrosis and changes in the composition and structural organization of the cartilage [2]. Unfortunately, clinical adoption remains limited due to the absence of standardized scanning and analysis protocols, robust validation, and reliable differentiation between healthy and OA cartilage [5].

Simulations can be used to address these challenges by enabling system design, acquisition optimization and validation of analysis techniques by providing ground truth when in vivo ground truth is unavailable [6]. Common US simulation tools, such as Field II, are based on linear wave propagation in homogeneous media, which limits their ability to capture complex wave phenomena [7,8]. Numerical wave solvers, such as finite-difference time-domain methods or pseudospectral methods, enable more accurate modeling of wave propagation in heterogeneous media, including scattering and diffraction [9,10]. Realistic US simulations, however, require both accurate wave modeling and detailed tissue phantoms that capture the tissue’s microstructure, as speckle patterns and intensity distributions strongly depend on the underlying tissue composition. While tissue phantoms with realistic microstructural detail can be generated from histological images, such approaches require histological data and extensive image segmentation [6]. For cartilage applications, several acoustic modeling approaches have been reported that do not rely on histological image inputs [11,12,13,14]. However, these existing acoustic cartilage models primarily focus on the superficial surface, assume homogeneity and isotropy, or include limited depth-dependent properties.

Therefore, this study aims to develop a simulation workflow using the k-Wave toolbox to generate US data of healthy and OA cartilage, incorporating literature-based depth-dependent acoustic and structural properties together with chondron-based microstructural scattering. Using a flexible, parameter-driven modeling strategy, the proposed framework enables the generation of realistic cartilage phantoms without the need for histological image-based model construction. This enables controlled adjustment of structural and acoustic tissue properties. In the present study, this capability was used to create cartilage models representing different stages of OA by adjusting tissue properties associated with cartilage degeneration. Furthermore, the flexible framework provides a platform for future studies investigating how individual tissue characteristics contribute to specific US features. In addition, such a framework can support the optimization of imaging and acquisition setups and can be readily extended to include other OA-related tissues. Ultimately, such models could contribute to the development of US-based diagnostic techniques, including data-driven and AI-based approaches for early detection, classification, and prediction of OA.

## 2. Materials and Methods

### 2.1. Simulation Workflow

**Figure 1:**
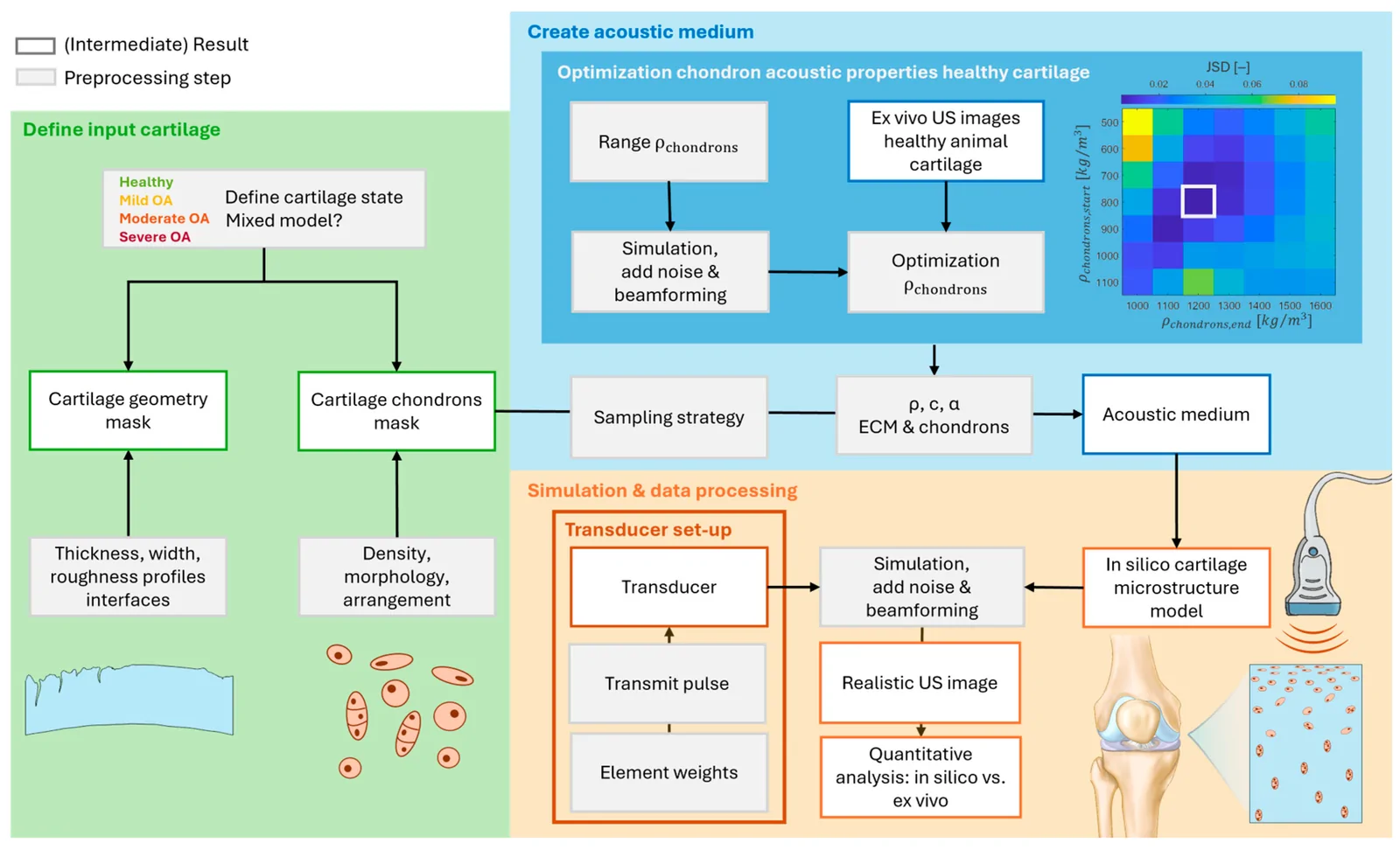
Workflow for in silico ultrasound (US) imaging of healthy and osteoarthritis (OA) cartilage. In green, the definition of the input cartilage and chondrons. The cartilage mask defines tissue morphology (thickness, width and surface roughness), whereas the chondron mask defines the spatial distribution, morphology, and density of chondrons. In blue, the construction of the acoustic medium. Chondron density ($\rho_{chondrons}$) was optimized using ex vivo samples, with the Jensen–Shannon Divergence (JSD) of US image gray-value distributions as the dissimilarity metric. The JSD plot shows the parameter sweep used for optimization of the healthy cartilage model, in which linearly varying depth-dependent chondron acoustic properties were evaluated. In orange, simulation to obtain US data, beamforming and further processing. Abbreviations: density (ρ), sound speed (c), acoustic absorption (α).

To enable the generation of realistic US images of healthy and OA cartilage without relying on histological image input, a parameter-driven workflow was developed; see Figure 1. The workflow combines depth-dependent tissue properties and chondron-based scattering. It consists of three main steps: (1) input cartilage definition, (2) acoustic medium generation and (3) US simulation and data processing. Cartilage is represented as an ECM embedding chondrons modeled as acoustic scatterers, with depth-dependent acoustic properties assigned to both components. In addition to healthy cartilage, the model includes three distinct stages of OA: mild, moderate, and severe, corresponding to input parameter ranges inspired by Mankin score subsets (1–3, 4–6, and 7–10), a commonly used histological grading system for cartilage degeneration. The associated structural and acoustic properties were varied systematically across these stages. Mixed models can also be generated, combining healthy and OA regions to reflect the heterogeneous nature of cartilage degeneration.

Ex vivo data from six healthy bovine and two OA human samples were used for optimization and evaluation of the workflow. US data were acquired using the Vevo F2 imaging system (FUJIFILM Visualsonics, Toronto, ON, Canada), equipped with a UHF29x linear array transducer (20 MHz center frequency, 15–29 MHz bandwidth), aligned above the superficial surface. Human cartilage samples were collected during knee arthroplasty procedures at Máxima Medical Center Veldhoven, The Netherlands, under institutional ethical approval and informed consent. Bovine cartilage samples were obtained from a local butcher in compliance with local regulations.

### 2.2. Defining the Input Cartilage

Cartilage input definition corresponds to the green block in Figure 1. The framework generates binary masks of the cartilage geometry and the chondrons based on input cartilage size, interface roughness, and depth-dependent chondron arrangement, density, and morphology, eliminating the need for histology-based segmentation [6]. The cartilage superficial surfaces were generated using a stochastic roughness model, in which 1D surface profiles were generated and scaled to match target roughness values sampled from the literature-reported ultrasound roughness index (URI) distribution. The URI quantifies cartilage surface roughness based on ultrasound backscatter and is 7.4 ± 1.2 µm for cartilage with a Mankin score of 0 [15,16].

**Figure 2:**
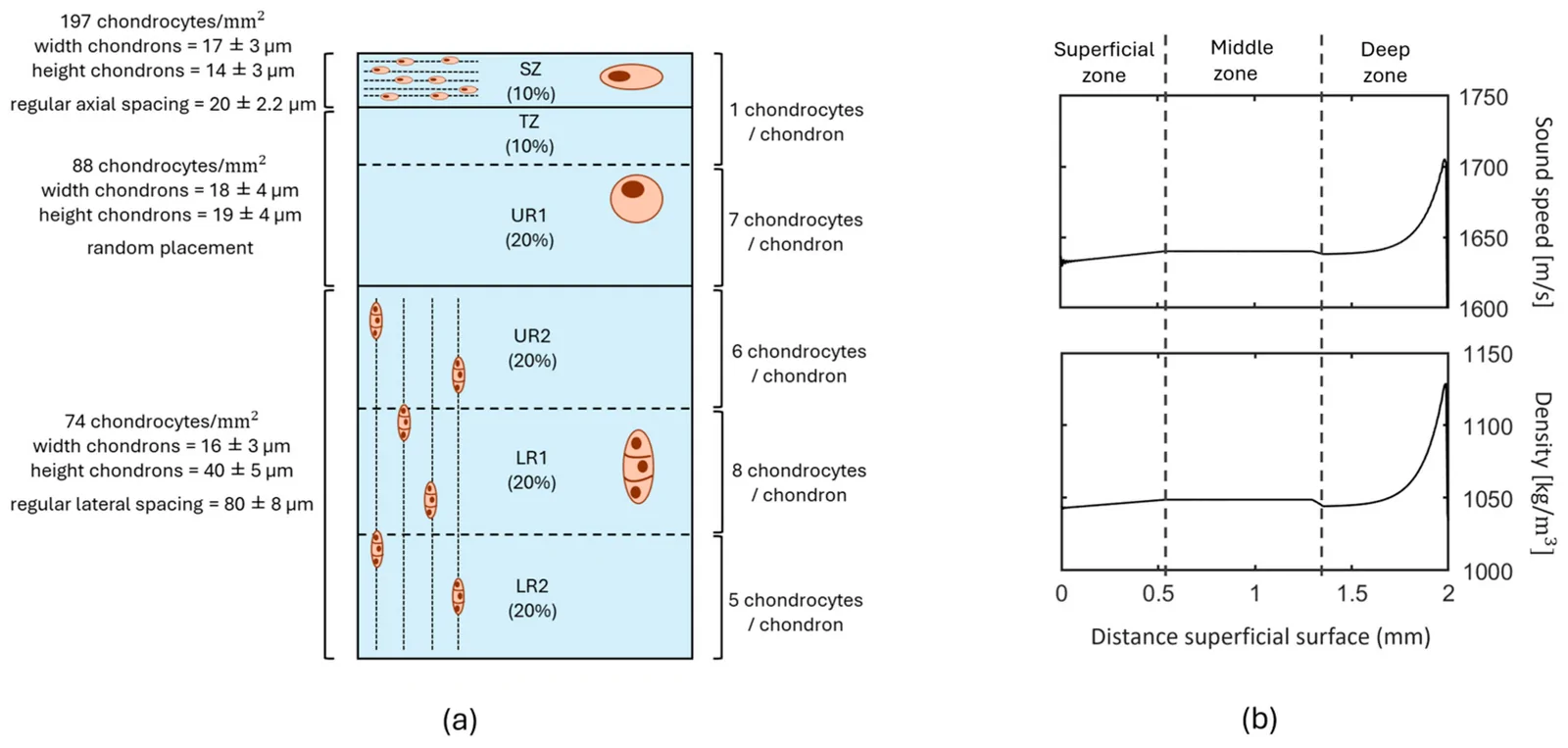
(**a**) Chondron definition of in silico healthy cartilage. The cartilage consists of six zones with distinct chondron density, shape, orientation, and arrangement. (**b**) Depth-dependent sound speed and density of the in silico ECM of healthy cartilage. Abbreviations: superficial (SZ), transitional (TZ), upper radial 1 (UR1), upper radial 2 (UR2), lower radial 1 (LR1), and lower radial 2 (LR2).

Figure 2a illustrates the in silico representation of chondrons in healthy cartilage, following depth-dependent density, morphology, orientation, and spatial arrangement typical of mature human tissue. The cartilage was divided into six zones: superficial (SZ, 10%), transitional (TZ, 10%), upper radial 1 (UR1, 20%), upper radial 2 (UR2, 20%), lower radial 1 (LR1, 20%), and lower radial 2 (LR2, 20%) [17]. From the average zonal chondrocyte densities (chondrocytes/mm^2^) and chondrocytes per chondron for the human knee [18,19], the zonal chondron densities (chondrons/mm^2^) were derived. Zonal chondron size and shape were based on values reported for the human knee by Choi et al. [20]. Moreover, in SZ the ellipsoid chondrons are oriented parallel to the superficial surface with a regular axial spacing of 20.8 µm and an allowed deviation of 2.2 µm, while their lateral positions are assigned randomly [14]. In TZ and UR1, the circular chondrons are randomly distributed. In UR2, LR1 and LR2, the column-like chondrons are oriented perpendicular to the superficial surface with a regular lateral spacing of 80 µm [21] and an allowed deviation of 8 µm, while their axial positions are assigned randomly.

**Figure 3:**
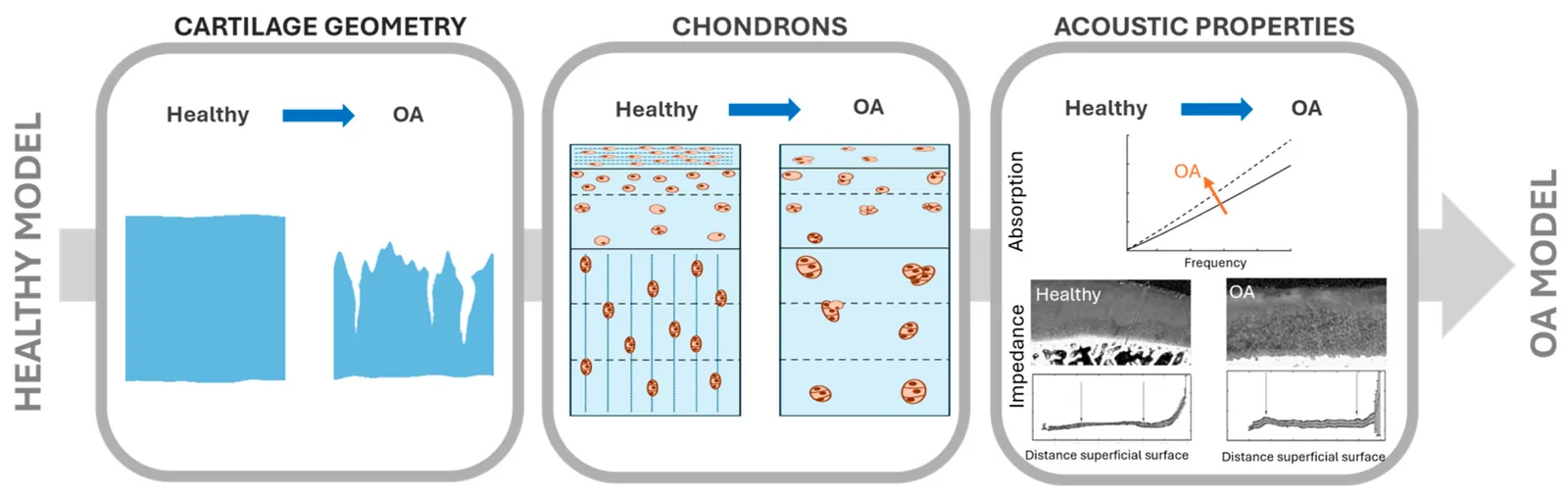
Changes in cartilage geometry, chondrons and acoustic properties with OA severity: surface roughening, adding superficial clefts, enlarging or removing chondrons and disrupting their spatial arrangement, increasing absorption, and changing impedance of the ECM according to [24].

Figure 3 shows progressive changes in cartilage geometry, chondron characteristics, and acoustic properties from healthy to severe OA, with input parameters detailed in Appendix B. Early OA may cause slight cartilage swelling, followed by thinning as it progresses [2,22]. Accordingly, a change in cartilage thickness was introduced into the OA models. Like healthy cartilage, OA tissue was tuned to match literature-reported URI values. A reference study reports URI values of 15.1 ± 8.5 µm for cartilage with Mankin scores 1–3 and 34.3 ± 15.4 µm for cartilage with Mankin scores 3–10 [16]. These values were used as reference points to tune the 1D superficial surface profiles for the modeled OA categories, representing progressive surface degeneration. Moreover, Mankin’s surface structure score shows increasing surface damage with higher scores: 1 = surface irregularities; 2 = pannus/fibrillation; 3 = clefts into the transitional zone; 4 = clefts into the radial zone; 5 = clefts reaching the calcified cartilage [23]. Accordingly, superficial fissures were introduced for mild OA, whereas deeper clefts were used for the moderate and severe OA models.

Furthermore, chondrocyte hypertrophy, clustering, and apoptosis characterize OA progression [25,26,27]. Superficial chondrocytes become hypertrophic, then apoptotic, and in severe OA, enlarged cells are evenly distributed throughout the cartilage [25,28]. Chondrocytes also lose spatial organization and remodeling ability, with early lesions showing irregular clusters and emerging new patterns [29]. Therefore, the framework incorporates progressive, depth-dependent changes in chondron size, density, and spatial organization in OA tissue. For mild OA, chondrons in the SZ and TZ are enlarged and randomly arranged, replacing the periodic axial spacing seen in healthy cartilage. In moderate and severe OA, all chondrons are enlarged and randomly distributed.

### 2.3. Creating the Acoustic Medium

Next, the acoustic medium is generated, as illustrated by the blue block in Figure 1. The masks are zero-padded, smoothed, low-pass filtered, and down-sampled onto the simulation grid to minimize staircasing errors [30]. Subsequently, acoustic properties are assigned to ECM and chondrons. The surrounding medium was defined as water.

Cartilage shows a depth-dependent acoustic impedance gradient, with a linear increase from the superficial to the middle zone, stability in the middle, and an exponential rise in the deep zone [24]. Given this, a depth-dependent speed-of-sound and density with mean values of 1640 m/s and 1050 kg/m^3^ [31,32] are prescribed to the in silico ECM of healthy tissue; see Figure 2b. Because separating tissue attenuation into individual components is challenging, absorption was fixed, and chondron scattering was optimized to match ex vivo US measurements. For healthy tissue, the absorption was set to 0.2 dB/mm/${MHz}^{1.1}$, corresponding to approximately 5.4 dB/mm at 20 MHz, which is consistent with measured values of 2.8–6.5 dB/mm for bovine cartilage in the frequency range of 10–40 MHz [33].

As illustrated in Figure 3, OA involved changes in ECM impedance and cartilage absorption. Damaged cartilage exhibits greater impedance variation and altered layer proportions compared to healthy tissue, due to loss of depth-dependent organization [24]. Specifically, the regions characterized by a linear increase in impedance from the superficial surface and an exponential increase towards the cartilage–bone interface become smaller, while the intermediate region with relatively uniform impedance becomes proportionally larger. Based on the depth-dependent impedance distributions reported for normal cartilage and cartilage with a Mankin score of 7, relative layer proportions were estimated for the mild, moderate, and severe OA models. These estimated layer proportions were subsequently used to generate the corresponding depth-dependent density and speed-of-sound profiles. Moreover, proteoglycan loss and collagen disruption increase frequency-dependent attenuation by 20–30% [34], so absorption was slightly raised in SZ and TZ for mild OA and further across all layers for moderate and severe OA. To represent progressive degeneration across the modeled OA stages, attenuation was increased by 10%, 20%, and 30% relative to healthy cartilage for the mild, moderate, and severe OA models, respectively. The 10% increase for mild OA was included as a modeling assumption to represent an earlier stage of degeneration. Chondrons retained the optimized acoustic properties of the healthy tissue, only adjusted for cartilage thinning.

#### Optimizing Chondron Acoustic Properties

**Figure 4:**
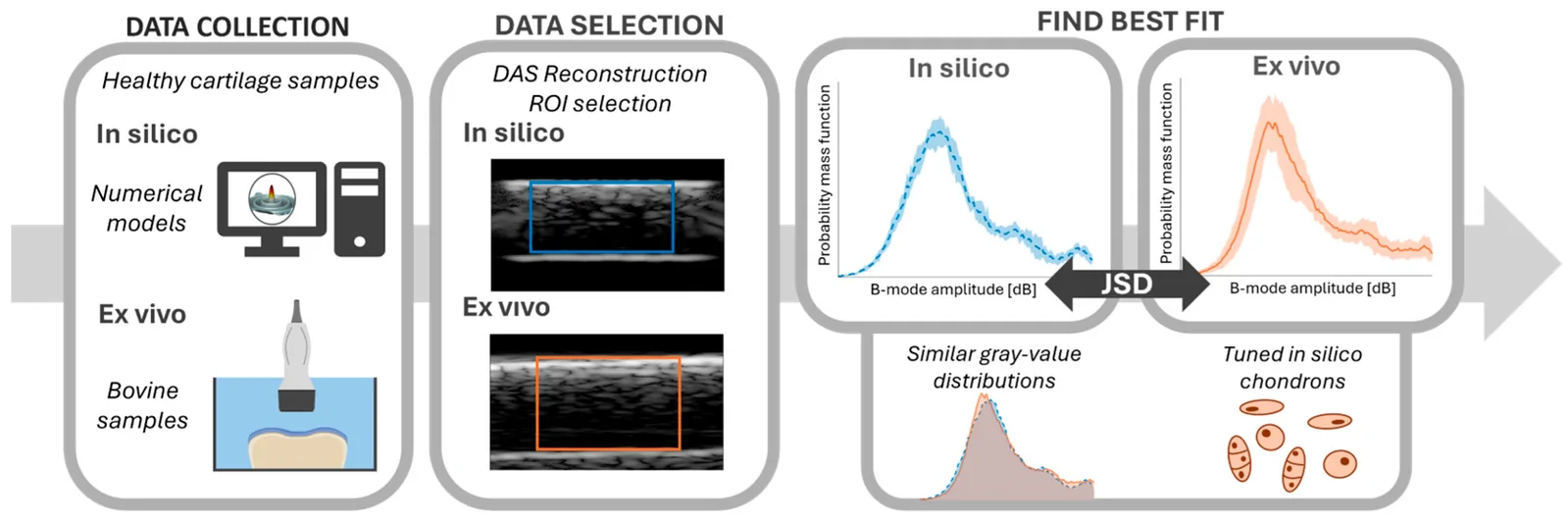
Optimization of chondron acoustic properties. A region of interest (ROI) is extracted from each US image, and measured and simulated gray-value distributions are compared using the Jensen–Shannon Divergence (JSD). The figure shows the healthy ex vivo data and the best-fitting simulation used for model calibration and evaluation. Blue-colored ROI and gray-value distribution correspond to the in silico data, whereas orange-colored ROI and gray-value distribution correspond to the ex vivo data. Abbreviations: delay-and-sum (DAS), region of interest (ROI).

The workflow used to optimize the chondron acoustic properties is shown in Figure 4. Preliminary results comparing models with only Gaussian fluctuations in acoustic properties, chondrons with uniform acoustic properties, and chondrons with depth-dependent acoustic properties indicated that linearly varying depth-dependent chondron acoustic properties were most suitable for generating realistic US images (see Appendix C). Therefore, simulations of healthy cartilage models (2 mm thick, 5 mm wide) were performed with a fixed chondron sound speed of 1500 m/s and depth-dependent chondron density profiles. A series of linear density profiles was evaluated, with densities ranging from 1000 to 2000 kg/m^3^ at both cartilage interfaces in increments of 100 kg/m^3^. Ten independent simulations were performed for each profile. To identify the best-fitting density profile, the simulated and ex vivo gray-value distributions were compared. Ex vivo data were acquired from six bovine tibia and patella cartilage–bone samples with intact surfaces and a thickness between 1.5 and 2.5 mm. All data were beamformed, and in each US image, regions of interest (ROIs; 3.7 mm wide, variable height) were defined, including the superficial reflection but excluding the second interface. Each simulated image included one ROI, while measured images featured multiple, totaling 25 ROIs. Data within each ROI were normalized to the 99th percentile and binned (0.55 bin width) using the Freedman–Diaconis rule [35] applied to the ex vivo data. The mean gray-value distributions were then calculated for the ex vivo dataset and for each simulated density profile. Finally, the in silico and ex vivo distributions were compared using the JSD [6,36]:

(1) $$JSD\left(P\|Q\right)=\frac{1}{2}\left(D_{KL}(P\|M)+D_{KL}(Q\|M)\right),$$

(2) $$M=\frac{1}{2}\left(P+M\right),$$

where P and Q are the discrete probability mass functions and $D_{KL}$ is the Kullback–Leibler Divergence, which is defined as [37]:

(3) $$D_{KL}\left(P\|M\right)=\sum_{i}P\left(i\right){log}_{2}\left(\frac{P\left(i\right)}{M\left(i\right)}\right)$$

This yields JSD values between 0 and 1, with lower values indicating greater similarity between simulated and measured US images. The depth-dependent chondron density that produced the lowest JSD (JSD = 0.004) is adopted in the model, ranging from 800 kg/m^3^ at the superficial surface to 1200 kg/m^3^ at the second cartilage interface. Only increasing profiles were considered, since deeper chondrons contain multiple chondrocytes and more pericellular matrix, whereas superficial chondrons contain a single chondrocyte.

### 2.4. Simulation and Data Processing

Once the acoustic medium is defined, simulations and data processing are performed, as shown in the orange block of Figure 1. The propagation of the acoustic waves is simulated over time according to the linear wave equation using the k-Wave toolbox version 1.4 [9] in MATLAB version 2024b. First, the UHF29x US transducer was defined by its transmit and receive elements, pitch, element width, center frequency, bandwidth, sampling frequency and transmit apodization. Its position and orientation were matched to the experimental setup used for the ex vivo measurements. To minimize staircasing errors during discretization, an analytical band-limited representation of the transducer element shape was applied [6,30]. Elements acted as velocity sources, transmitting normal to their surface with a single-frequency tone burst, while channel data were acquired using a zero-degree plane wave in the transducer’s 2D imaging plane. The transducer implementation was evaluated by comparison of in vitro and in silico speckle sizes obtained from a scattering phantom; see Appendix A. Using 10 points per wavelength at the transducer center frequency resulted in a grid spacing of 7.5 µm. The computational domain extended 30 mm in the lateral direction and 10 mm in the axial direction, with a perfectly matched layer of 20 grid points applied at the domain boundaries. A CFL number of 0.3 and a simulation time of 25 µs were used.

Gaussian white noise was added to the simulated signals, mimicking electrical noise, with power estimated from in vitro measurements as a fraction of the dynamic range. The system’s frequency response was modeled with a Hann window in the temporal frequency domain, using the transducer’s center frequency and −6 dB bandwidth [30]. The same filter was also applied to all ex vivo data to suppress noise and out-of-band artifacts before reconstruction. All data were delay-and-sum beamformed to create B-mode US images. The pixel spacing used for the reconstruction grids was 18.75 µm, corresponding to 4 points per wavelength. For beamforming, a reference sound speed of 1640 m/s, an apodization angle of 15 degrees, and a receive apodization using a Hann window were used.

### 2.5. Quantitative Analysis

To quantitatively compare in silico cartilage damage states (healthy, mild OA, moderate OA, severe OA) and against ex vivo samples, commonly used US metrics reported in the literature were extracted. These metrics were obtained from time gates at the cartilage surface (surface parameters) and within the cartilage matrix (backscatter parameters) (final analysis step in Figure 1, orange block). Ten simulations were conducted for each cartilage state using identical acoustic parameters but different microstructural configurations, including variations in chondron placement and cartilage geometry realization.

US roughness index (URI), reflection coefficient (R), and integrated reflection coefficient (IRC) were calculated following the approach described in [4,38]. URI (µm) was computed from the cartilage surface profile obtained by detecting the water–cartilage interface, followed by high-pass filtering [4,38]. R (%) was calculated as the ratio of the peak-to-peak amplitude of the cartilage surface reflection to that of a water–aluminum reference interface at the corresponding distance. For IRC (dB), amplitude spectra of the cartilage reflection and the reference signal were computed using FFT after zero-padding the windowed signal to 4096 points to improve frequency resolution [38]. Depth-dependent profiles of backscatter amplitude (apparent integrated backscatter, AIB [4]) were used to estimate the attenuation in dB/mm. AIBslope was estimated from depth-dependent AIB profiles obtained via short-time Fourier transform (STFT) of gated radiofrequency signals within the cartilage matrix, followed by linear regression over the first millimeter below the superficial surface. Moreover, gray-value distributions of reconstructed US images were computed following the same procedure used for optimizing the chondron acoustic properties of the healthy model, described in Section 2.3 under Optimizing Chondron Acoustic Properties. It should be noted that the healthy cartilage gray-value distributions used for model evaluation were identical to those used to optimize the model’s depth-dependent chondron density. All parameters were averaged across transducer elements positioned above the cartilage: the middle 50 elements were used for simulations, whereas all 256 elements were included for measurements.

## 3. Results

**Figure 5:**
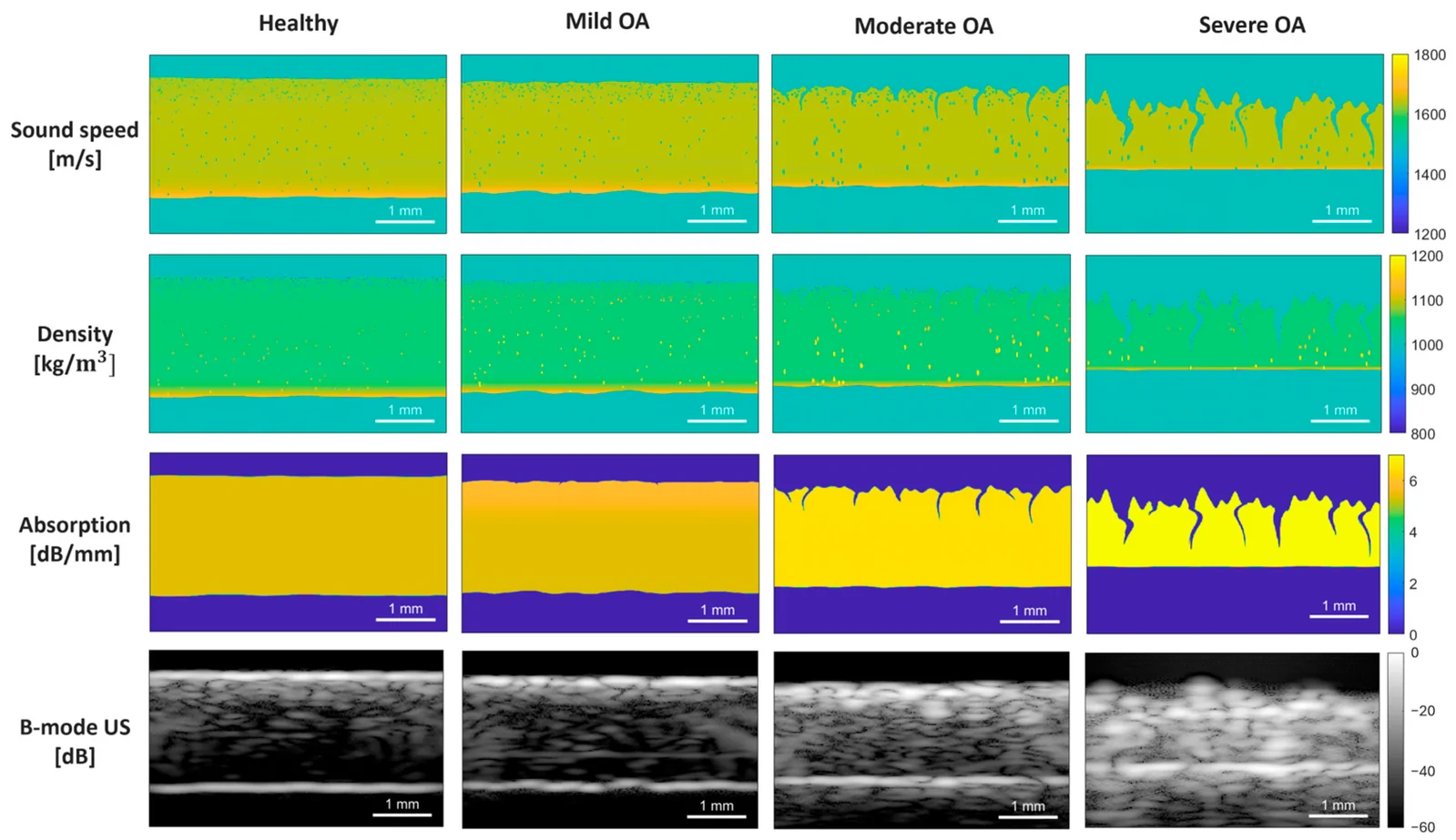
Examples of sound speed (m/s), density (kg/m^3^), and absorption (dB/mm) distributions, along with reconstructed US images for in silico healthy, mild OA, moderate OA, and severe OA cartilage states.

**Figure 6:**
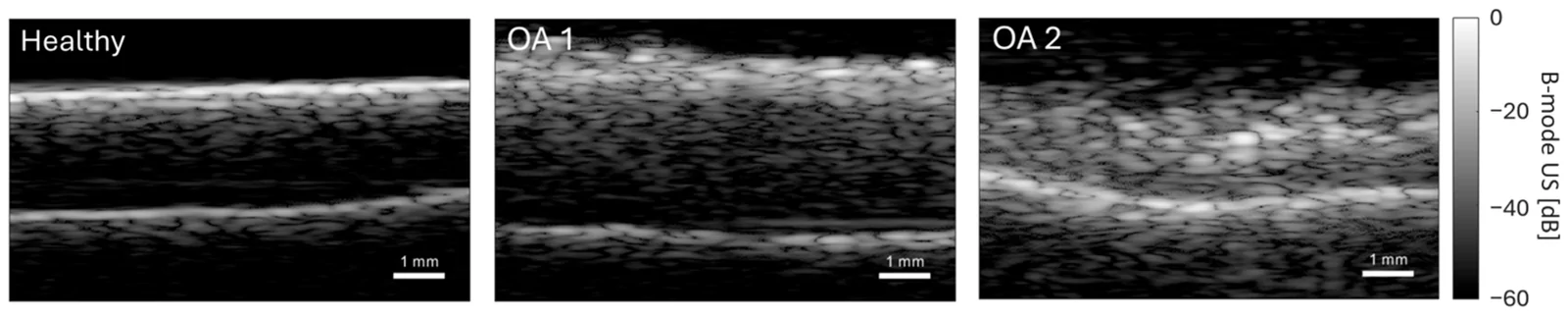
Ex vivo US images: healthy bovine tibial cartilage (Healthy), OA human tibial cartilage with visible surface irregularities (OA 1), and OA human tibial cartilage exhibiting pronounced structural damage (OA 2).

Example input distributions of density (kg/m^3^), sound speed (m/s) and absorption (dB/mm) together with reconstructed US images of the in silico cartilage states are shown in Figure 5. The resulting cartilage models (including both the ECM and chondrons) exhibited density and speed-of-sound ranges of 800–1200 kg/m^3^ and 1630–1720 m/s, respectively. Figure 6 shows ex vivo US images of healthy and OA cartilage. Normal cartilage appears as a uniformly anechoic layer with sharp, continuous interfaces. OA is characterized by decreased superficial echogenicity, increased echogenicity at the cartilage–bone interface, loss of the homogeneous anechoic appearance, surface irregularities, and focal or asymmetric cartilage thinning that can progress to complete erosion [15]. Figure 5 demonstrates that the simulated US images reflect these features, showing a clear trend of increasing severity from healthy to mild, moderate, and severe OA.

**Figure 7:**
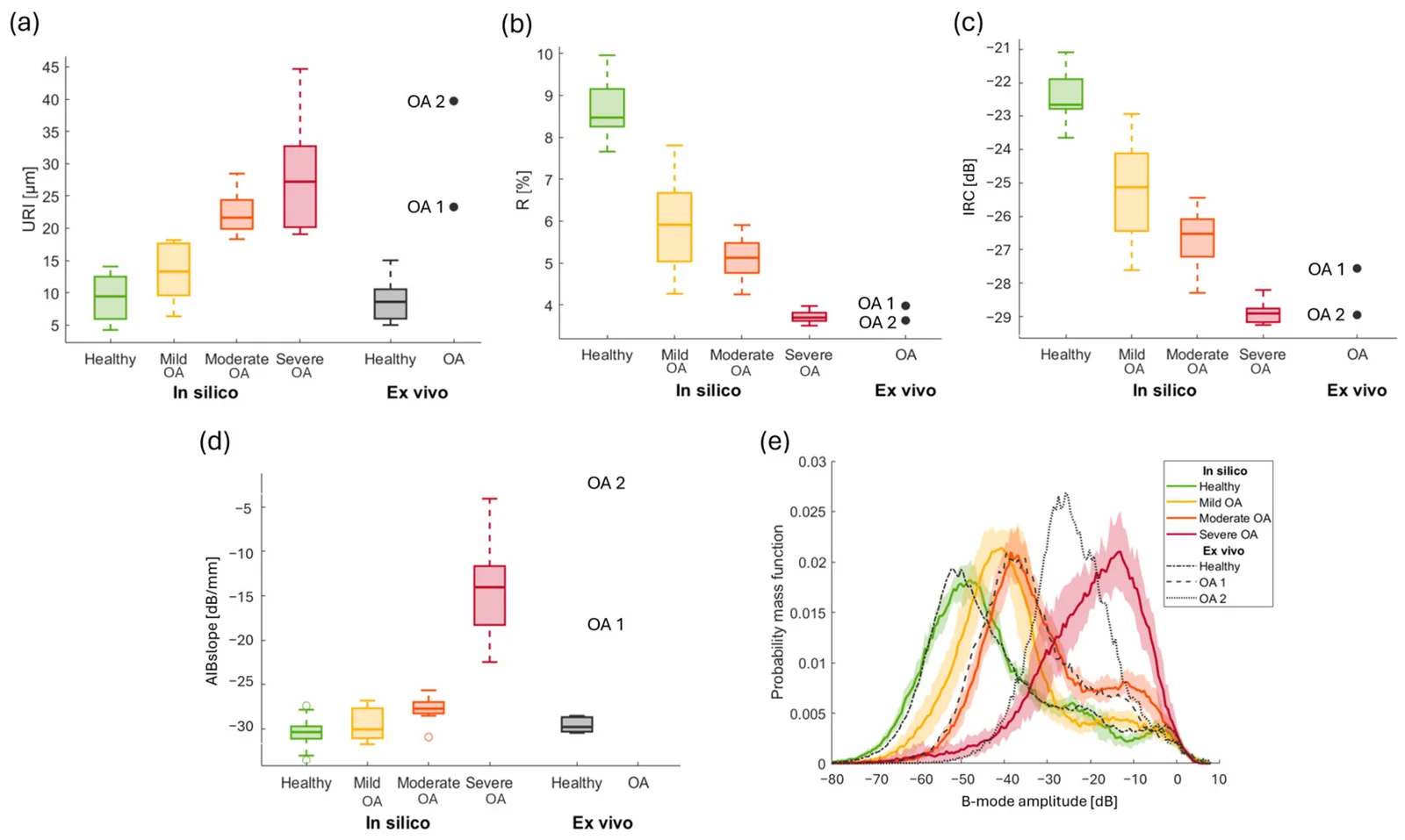
Surface parameters (URI, R, IRC) and backscatter analysis (gray-value distributions and attenuation over depth, AIBslope) between the in silico cartilage states and ex vivo healthy and OA samples. The healthy data are from the bovine samples used for optimization; the OA data are from the two samples in Figure 6. Subplots: (**a**) URI, (**b**) R, (**c**) IRC, (**d**) AIBslope, and (**e**) gray-value distributions (probability mass function vs. B-mode amplitude).

Quantitative US metrics were computed for all in silico cartilage states and the ex vivo samples. The surface parameters, URI, R and IRC for all cases of the model and the ex vivo data, are shown in Figure 7a–c. All reported values are medians. From healthy to severe OA, the superficial surface exhibited increased roughness and reduced reflection capacity. In the model, URI increased from 9.44 µm to 27.21 µm, R decreased from 8.47% to 3.70%, and IRC decreased from −22.66 dB to −28.91 dB. Likewise, in the ex vivo samples, URI increased from 8.58 µm in healthy cartilage to 23.30 µm and 39.71 µm for the two OA samples. Reflection coefficients could not be computed for the ex vivo healthy bovine cartilage samples, as some transducer elements were saturated due to the gain and transmit-pulse voltage settings.

The AIB slope, reflecting the frequency-dependent attenuation, also showed some differences between healthy and OA tissue; see Figure 7d. In the model, the AIB slope increased from −30.37 dB/mm to −14.05 dB/mm from healthy to severe OA. In the ex vivo samples, it increased from −29.80 dB/mm to −18.02 dB/mm and −1.98 dB/mm. Moreover, the distributions of gray values of the US images show clear differences between healthy and OA cartilage; see Figure 7e. Good agreement is observed for healthy cartilage, for which the gray-value distributions shown in Figure 7e were the same as those used for optimization of the healthy model. From healthy to severe OA, a rightward shift, indicating higher echo amplitudes, and a narrower distribution are observed. Notably, especially for healthy cartilage, the distribution deviates from a Gaussian shape due to its complex composition and structural organization. This highlights the need to incorporate detailed structural and acoustic properties into the in silico model to accurately represent cartilage microstructure (supported by the gray-value distributions shown in Appendix C).

**Figure 8:**
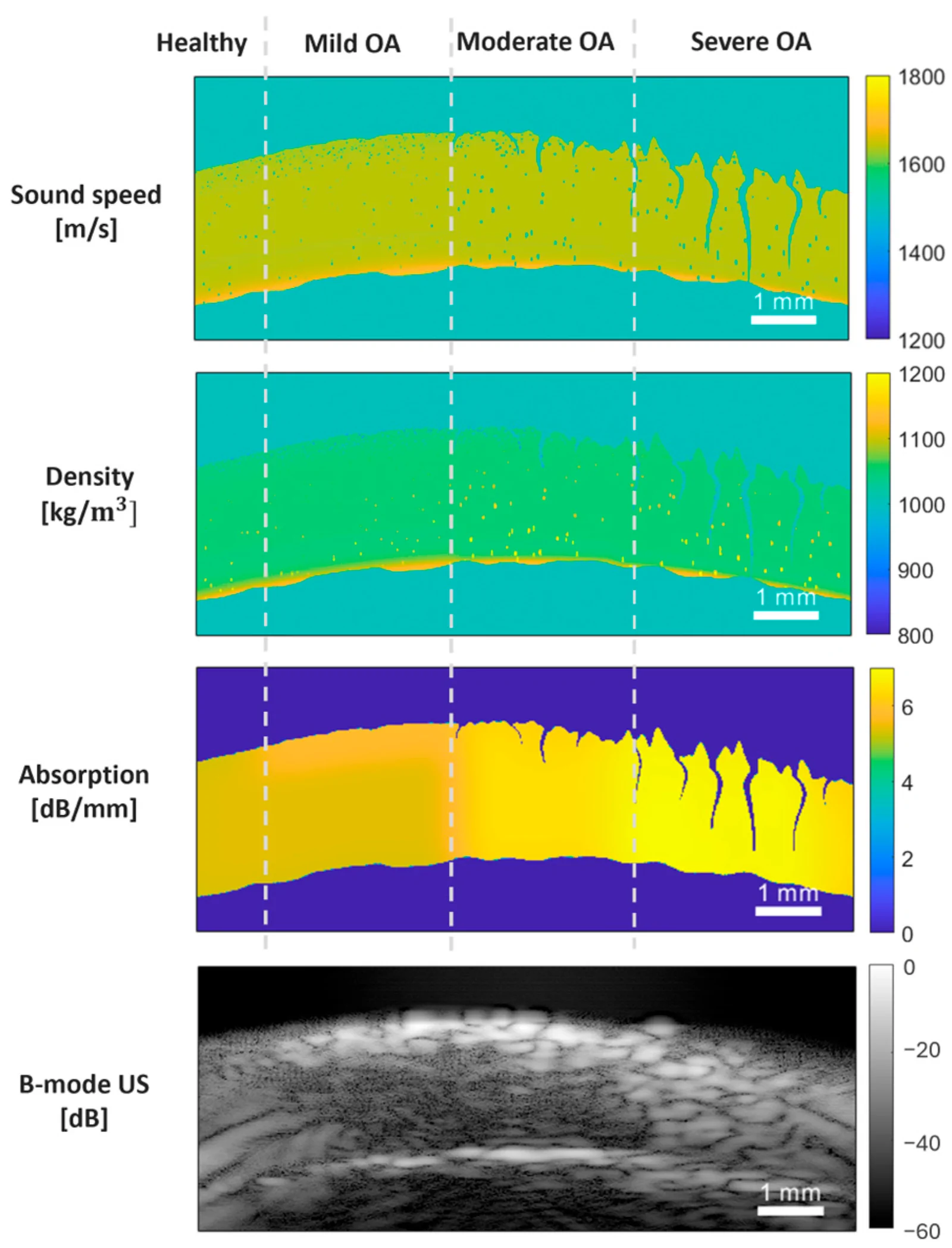
Sound speed (m/s), density (kg/m^3^) and absorption (dB/mm) distributions along with the reconstructed B-mode US image of a mixed cartilage model consisting of 10% healthy, 30% mild OA, 30% moderate OA, and 30% severe OA tissue.

An example of the input distributions and the corresponding US image of a mixed model, consisting of approximately 10% healthy, 30% mild OA, 30% moderate OA and 30% severe OA tissue divided into four segments, is shown in Figure 8.

## 4. Discussion

A simulation framework for US imaging of healthy and OA cartilage was developed and evaluated. The acoustic cartilage model incorporates chondrons as scatterers, and depth-dependent properties, and represents different damage stages, from healthy cartilage to severe OA. Due to the detailed maps of sound speed, density, and absorption, the framework generates US images (Figure 5 and Figure 8) that closely resemble real US measurements (Figure 6). In addition to visual similarity, quantitative US metrics were compared with the available ex vivo data and literature-reported values, demonstrating that the model captures the main trends associated with cartilage degeneration (Figure 7).

OA cartilage no longer exhibits a uniform anechoic appearance but shows increased echogenicity due to surface and internal degeneration, including reduced water content and fibrous changes [2]. These features are also observed in the simulated US images, indicating that the model captures key characteristics associated with cartilage degeneration. Similar observations can be made from the gray-value distributions: healthy cartilage showed a broad distribution due to its layered organization, whereas OA cartilage exhibited narrower distributions with higher B-mode amplitudes, suggesting a more homogeneous echogenic pattern. As shown in Appendix C, Gaussian fluctuations in sound speed and density or chondrons with uniform acoustic properties cannot reproduce the characteristic gray-value distribution associated with the layered organization of healthy cartilage. In contrast, the model’s chondron representation and depth-dependent properties enable a more realistic representation. Notably, the OA sample with less visible damage (OA1) showed the greatest similarity to the simulated moderate OA case, whereas the more visibly damaged sample (OA2) showed characteristics intermediate between the simulated moderate and severe OA cases. As histological grading was not available for these specimens, these observations should be regarded as qualitative comparisons rather than validation of the assumed Mankin-based severity mapping. By mixing characteristics of different OA stages (mixed model), it is possible to create intermediate states that closely match such samples. Moreover, the observed differences between the gray-value distributions of healthy and OA tissue in the simulated datasets suggest that distribution shape and amplitude may contain information related to cartilage degeneration, which has not yet been explored to the best of our knowledge. However, further validation in a larger ex vivo dataset is needed to assess the potential value for cartilage damage characterization.

Quantitative US-derived metrics (URI, R, IRC) describing surface roughness and reflection were analyzed. Due to surface roughening and enzymatic degradation of the collagen network during OA progression [2], URI increases, while R and IRC decrease, which was also observed in the simulated data. In silico healthy tissue was approximated by a Mankin score of 0, mild OA by scores 1–3, moderate OA by scores 4–6, and severe OA by scores 7–10. Direct comparison of R and IRC with the healthy ex vivo data was not possible due to transducer saturation. URI values aligned well with previous studies, while R and IRC were slightly higher [15,16,39]. There are, however, also studies that report R and IRC values comparable to those observed in this study [4,40,41]. Additionally, AIBslope becomes less negative with OA progression, reflecting reduced frequency-dependent attenuation linked to changes in collagen organization and composition [2]. Although the model reproduced the trend towards less negative AIB slopes with increasing OA severity, the absolute values differed from those reported in the literature [40,42], while some discrepancies also remained between the in silico and ex vivo results. Only a few studies report AIBslopes for cartilage, and the reported values vary substantially, ranging from approximately −100 dB/mm in healthy cartilage reported by Lye et al. [40] to around −20 dB/mm reported by Männicke et al. [43]. These differences likely arise from variations in calculation methods and imaging setups, limiting direct comparison of AIBslope values.

The model relies on numerous literature-derived parameters originating from different studies, species, and anatomical sites, whose limited availability and variability introduce uncertainty into the model assumptions and resulting simulations. In addition, the influence of individual model parameters remains unclear, as no formal sensitivity analysis was performed. Moreover, two compromises were made: (i) fixing absorption and optimizing scattering to match attenuation of the healthy cartilage model, as separating these effects is challenging, and (ii) optimizing this scattering using bovine samples, due to the limited availability of healthy human cartilage. Bovine cartilage differs structurally and acoustically from human tissue [18,34]. Differences in chondron morphology and density and acoustic properties affect the scattering and hence the optimized density of the chondrons. Furthermore, because the model is two-dimensional, it does not account for scattering contributions in the elevation direction. Consequently, the optimized chondron density should be interpreted as an effective model parameter rather than a true tissue property. Notably, the parameter sweeps showed that moderate variations in depth-dependent chondron density resulted in only minor changes in JSD (see Figure 1), whereas models lacking depth-dependent chondrons as scatterers showed poorer agreement (Appendix C). Moreover, cartilage exhibits substantial site-to-site and joint-to-joint biological variability, meaning that tissue properties can vary considerably even within a single species [18]. In addition, the model does not explicitly include a collagen network, but instead represents the ECM using effective, depth-dependent acoustic properties.

The depth-dependent acoustic impedance assigned to the chondrons was optimized using the healthy ex vivo dataset based on gray-value distributions. Gray-value distributions were used for this optimization as the chondron–ECM impedance contrast directly influences the resulting speckle intensity distribution. While gray-value distributions capture only first-order intensity statistics, complementary texture- and correlation-based metrics may provide additional information for model evaluation. Note that the healthy ex vivo dataset was used both for optimization of the chondron acoustic impedance and for evaluation of the resulting gray-value distributions. As a result, the JSD agreement obtained for healthy cartilage should be interpreted in the context of this optimization rather than as a fully independent validation. Furthermore, the healthy cartilage model was evaluated using six bovine samples, while the OA models were evaluated against two human ex vivo samples. This proof-of-concept study aimed to demonstrate the feasibility of modeling both healthy and OA cartilage using a simulation framework that incorporates detailed cartilage microstructural features. Further refinement and validation of the OA model will require a larger, histopathologically characterized dataset spanning multiple OA severity stages.

In addition, future work will focus on extending the model by coupling it with an optical model to enable photoacoustic simulations. Wu et al. [44] have shown that photoacoustic imaging with multiple wavelengths enables characterization of cartilage composition, revealing collagen degradation with distinct spectral differences across cartilage damage levels. Furthermore, this framework can be extended to include additional joint tissues affected during OA progression. The framework enables controlled variation of individual structural and acoustic parameters to investigate their effects on the US signal, which is difficult to achieve experimentally and may improve the interpretation of clinical US data. Ultimately, the model may aid in the generation of realistic synthetic datasets for optimizing imaging setups and developing AI-driven approaches for cartilage damage classification.

## 5. Conclusions

In conclusion, this work introduces a simulation framework for generating realistic synthetic US data of both healthy and OA cartilage by combining a microstructural cartilage phantom with accurate wave modeling. The model reproduces key US features and captures trends associated with increasing cartilage degeneration that are consistent with observations in the literature and the limited ex vivo dataset. As a proof-of-concept study, these findings demonstrate the feasibility of using microstructure-based simulations to model both healthy and OA cartilage. Although further validation using a larger ex vivo dataset, together with assessment of parameter sensitivity and model generalizability, is required, the microstructural detail of the cartilage model provides a platform for the systematic investigation of cartilage properties in relation to US features. Furthermore, the framework can be used to generate synthetic US datasets that may support the future development and evaluation of data-driven and AI-based approaches for OA assessment and monitoring.

## Figures and Tables

**Figure A1:**
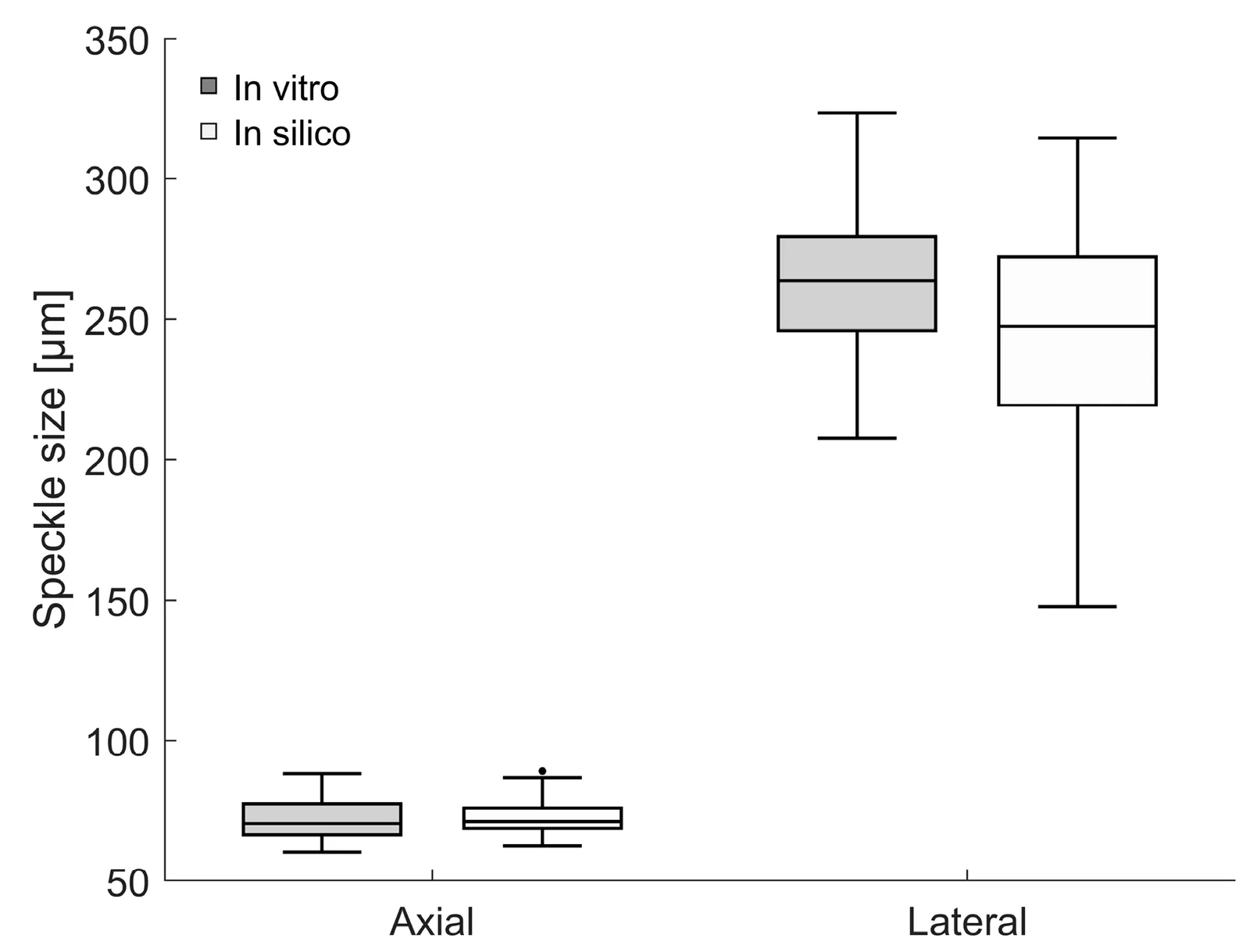
Speckle size distributions for the UHF29x linear array transducer acquired on a PVA (polyvinyl alcohol) phantom.

**Figure A2:**
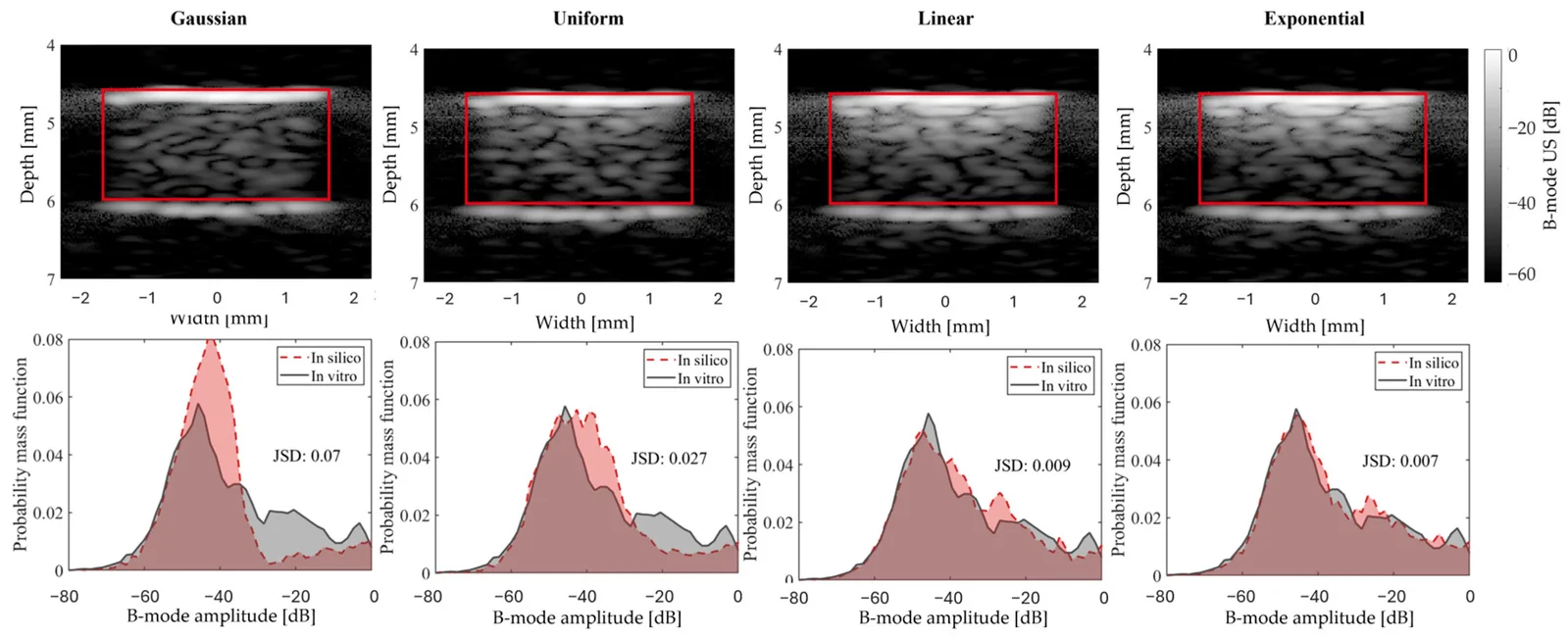
Comparison of ex vivo gray-value distributions and corresponding distributions obtained from preliminary in silico models (red) using different scatterer modeling approaches: best-fit Gaussian fluctuations in sound speed and density, uniform chondron acoustic properties, depth-dependent linear density variation with uniform sound speed, and depth-dependent exponential density variation with uniform sound speed.

## Data Availability

The original contributions presented in this study are included in the articl. Further inquiries can be directed to the corresponding author.

## Abbreviations

The following abbreviations are used in this manuscript:

AIB	Apparent integrated backscatter
AIBslope	Apparent integrated backscatter slope
DAS	Delay-and-sum
ECM	Extracellular matrix
IRC	Integrated reflection coefficient
JSD	Jensen–Shannon divergence
LR1	Lower radial 1
LR2	Lower radial 2
OA	Osteoarthritis
PCM	Pericellular matrix
R	Reflection coefficient
ROI	Region of interest
SZ	Superficial zone
TZ	Transitional zone
UR1	Upper radial 1
UR2	Upper radial 2
US	Ultrasound

## Appendix A

The transducer implementation was evaluated by analyzing the axial and lateral speckle sizes at various depths, as shown in Figure A1. The in vitro and in silico axial speckle sizes were comparable, measuring 72 ± 7 μm and 73 ± 6 μm, respectively. A difference of 20 μm can be observed between the in vitro and in silico lateral speckle sizes, which are 264 ± 30 μm and 244 ± 40 μm, respectively. This discrepancy was considered acceptable for the intended application and subsequent speckle-based calibration.

The in vitro phantom consisted of 50 wt.% demineralized water, 34 wt.% glycon ethylene, 12 wt.% PVA particles and 2 wt.% SiC. US imaging was performed with the UHF29x transducer, a single zero-degree plane wave and 64 active elements. The in vitro phantom’s sound speed was estimated using a global autofocusing method that maximized the speckle power by varying the reconstruction speed-of-sound [45], yielding 1540 m/s. An acoustic absorption coefficient of 0.0417 dB/MHz/mm was estimated using a linear fit to the intensity decay of the measured radiofrequency data. The in silico scattering phantom was described with a density of 1000 kg/m^3^, the estimated sound speed and absorption coefficient and a speckle intensity of $10^{-8}$ (unitless). The in silico and in vitro speckle sizes were determined by computing the normalized autocorrelation of the envelope data. Square-shaped ROIs measuring 1.5 by 1.5 mm with an 80% overlap in the axial direction were selected at various depths [36].

## Appendix B

The input parameter values used in this study for every case of the acoustic cartilage model are shown in Table A1.

**Table A1:** Overview of used input parameter values of the cartilage model for the mild OA, moderate OA and severe OA cases compared to the healthy reference.

			Healthy	Mild OA	Moderate OA	Severe OA
**Cartilage** **geometry**	Thickness difference, compared to healthy reference [%]	(mean ± SD)	-	0 ± 5	−10 ± 10	−30 ± 10
	Superficial cleft width [µm]	(mean ± SD)	-	30 ± 0	80 ± 20	150 ± 50
	Superficial cleft depth [%]		-	0–3	5–30	15–90
**Chondrons**	Size increase, compared to healthy reference [%]	SZ, TZ: UR1, UR2: LR1, LR2:	- - -	25 ± 10 0 0	80 ± 20 80 ± 20 80 ± 20	80 ± 20 80 ± 20 80 ± 20
	Elimination, compared to healthy reference [%]	SZ, TZ: UR1, UR2: LR1, LR2:	- - -	20 0 0	30 20 10	40 30 20
	Position/placement	SZ: TZ, UR1: UR2, LR1, LR2:	Regular axial spacing (20.8 ± 2.2 µm) Random Regular lateral spacing (80 ± 8 µm)	Random Random Regular lateral spacing (80 ± 8 µm)	Random Random Random	Random Random Random
**Acoustic** **properties**	Proportions of ρ/c of the ECM (linear increase, uniform, exponential increase) [%]		20, 41, 32	20, 50, 30	13, 67, 20	10, 75, 15
	Absorption coefficient [dB/mm/MHz^1^·^1^]		0.2	0.22 (Only applied to the upper 20% of the cartilage)	0.24	0.26

## Appendix C

To explore the mechanisms underlying the observed gray-value distributions, several preliminary modeling approaches were investigated during the early stages of this study. These simulations were performed using a histology-based framework in which a cartilage geometry and chondrons were segmented from a histological image of normal cartilage and assigned acoustic properties according to different scatterer models. For each approach, a single simulated ROI was compared with a corresponding ex vivo ROI, and only the best-fitting result is shown in Figure A2. The evaluated approaches included Gaussian fluctuations in sound speed and density, uniform chondron acoustic properties, and depth-dependent linear and exponential density variations with uniform chondron sound speed. The Gaussian and Uniform models resulted in the highest JSD values, while the Linear and Exponential models produced the lowest JSD values. For the Gaussian model, the probability mass function exhibited a higher peak than the in vitro measurement, reflecting a more uniform gray-value distribution within the simulated ROI. In addition, the Gaussian and Uniform models showed poor agreement with the right tail of the distribution, which corresponded to lower B-mode amplitudes in the superficial cartilage layer compared with the in vitro image. Overall, these simpler scatterer models, based on Gaussian fluctuations or uniform acoustic chondron properties, did not adequately reproduce the observed gray-value distributions, motivating further refinement towards the microstructural model presented in this study.
